# Ideal Protein-Based Estimation of Essential Amino Acid Requirements and The Role of Lysine Supplementation in Low-Protein Feed Formulations of Abalone

**DOI:** 10.1016/j.cdnut.2025.107528

**Published:** 2025-08-14

**Authors:** Yao-Bin Ma, Wei-Guang Zou, Chun-Xiang Ai, Sheng-Tai Liu, Xuan Luo, Wei-Wei You, Cai-Huan Ke

**Affiliations:** 1 Fujian Key Laboratory of Genetics and Breeding of Marine Organisms, College of Ocean and Earth Sciences, Xiamen University, Xiamen, China; 2 Animal Nutrition Institute, Sichuan Agricultural University, Chengdu, Sichuan, China; 3 Fujian Ocean Innovation Center, Xiamen, China

**Keywords:** abalone, low-protein diet, ideal protein, lysine supplementation, nutrient utilization

## Abstract

**Background:**

High dietary protein levels in aquafeeds lead to increased nitrogenous waste, which requires the development of sustainable feeding strategies that balance growth performance with environmental responsibility.

**Objectives:**

This study calculated the ratio of essential amino acids to lysine and investigated whether dietary protein could be reduced in feeds for Lvpan abalone (*Haliotis discus hannai*♀ × *Haliotis fulgens*♂) by lysine supplementation.

**Methods:**

Five diets were formulated to be isoenergetic: a control diet (CP27, 27% crude protein), 2 low-protein diets (CP23 and CP19, 23% and 19% CP, respectively), and their lysine supplementation counterparts (CP23 + AA and CP19 + AA). After an 84-d feeding trial (initial abalone weight, 19.49 ± 0.52 g), the growth, digestibility, nitrogen excretion, hepatic transaminases, muscle composition, water-holding capacity, texture, and volatile profile were measured.

**Results:**

The CP23 + AA diet maintained growth performance, improved apparent protein digestibility, and decreased nitrogen excretion compared with the control (*P* < 0.05). In contrast, the CP19 + AA diet reduced growth (*P* < 0.05). Lysine supplementation in the CP23 + AA group resulted in an increase in transaminase activity compared with the CP23 group, but it was not statistically significant (*P* > 0.05). Transaminase activity was greater in the CP19 + AA group than in the unsupplemented CP19 group (*P* < 0.05).

**Conclusions:**

A 4% reduction in dietary protein, when supplemented with lysine, demonstrates potential for sustainable abalone aquaculture through sustained growth and product quality coupled with reduced nitrogenous waste production.

## Introduction

Dietary protein constitutes a primary source of amino acids for sustaining essential life processes and promoting growth in aquatic animals [1]. The catabolism of protein for energy yields ∼16% of its nitrogen content as ammonia, urea, and other nitrogenous waste products [2]. Due to excessively high expectations of profitability among aquaculturists, the protein content in formulated aquatic feeds has been consistently maintained at high levels [3]. This practice results in adverse consequences, including elevated nitrogenous waste discharge into culture systems, deterioration of water quality, and increased vulnerability to disease outbreaks [4]. Low-protein diets present a dual advantage by not only reducing feed costs and enhancing protein utilization but also concurrently minimizing ammonia–nitrogen emissions. Nonetheless, reducing dietary protein levels can potentially introduce amino acid imbalances within the feed formulation, thereby compromising the normal growth and development of farmed aquatic species [5]. Numerous studies have demonstrated that the formulation of low-protein aquafeeds based on the ideal protein concept can enhance feed protein utilization, conserve protein resources, and improve economic profitability [[6], [7], [8]].

The rationale underpinning low-protein diet formulations is rooted in the ideal protein concept, initially articulated by Mitchell and Block in 1946 [9]. The significance of maintaining an appropriate amino acid balance is frequently illustrated by the barrel theory. Analogously, if each stave of a barrel represents a discrete amino acid, the overall capacity of the barrel is contingent upon the length of the shortest stave. In nutritional terms, this limiting component is designated the first-limiting amino acid [10]. According to the ideal protein concept, although the absolute requirements for individual essential amino acids in animals may fluctuate under varying feeding regimens, the relative ratios between these amino acids tend to remain comparatively constant [11]. Lysine is essential for protein synthesis and is often deficient in common feed ingredients, making it the first-limiting amino acid in many aquaculture species [8]. Consequently, when feeding conditions are altered, it is primarily necessary to determine the requirement for a single reference amino acid, typically lysine, as the requirements for other essential amino acids can be derived from the established ratios within the ideal protein profile [12]. Recent studies demonstrate that protein reduction (4%–8%) with limiting AA supplementation maintains growth in rainbow trout (*Oncorhynchus mykiss*) [13], grass carp (*Ctenopharyngodon idella*) [14], and tilapia (*Oreochromis niloticus* L.) [15].

Analogous to other teleost species, abalone cannot synthesize all essential amino acids de novo, thereby necessitating their dietary acquisition [16]. Diets deficient in essential amino acids can precipitate malnutrition and compromised growth performance in abalone [17]. It is well established that abalone require 10 essential amino acids [18]. Specifically, Allen and Kilgore [19] identified threonine, valine, methionine, isoleucine, leucine, phenylalanine, tryptophan, lysine, histidine, and arginine as essential amino acids for the red abalone (*Haliotis rufescens*). However, investigations into both the quantitative amino acid requirements and the ideal protein profiles for abalone remain scarce. Determining the precise quantitative requirements for individual essential amino acids in commercially farmed abalone species poses a considerable challenge. Fleming et al. [16], in their review of abalone amino acid nutrition, underscored the inherent difficulties in accurately ascertaining the specific requirements for individual amino acids. Coote [20] reported that lysine comprises 5% of the total soft tissue protein in greenlip abalone (*Hydrocharis laevigata*) and subsequently calculated the requirements for each amino acid in this species, utilizing lysine as the primary limiting amino acid.

Our research group has previously reported the successful development of a novel hybrid species, the Lvpan abalone (*Haliotis discus hannai♀ × Haliotis fulgens♂*), via interspecific hybridization. This involved the crossbreeding of green abalone (*H. fulgens*) as the paternal progenitor with Pacific abalone (*H. discus hannai*) as the maternal progenitor [21]. The resulting Lvpan abalone exhibit notable hybrid vigor, particularly in terms of growth performance and tolerance to environmental stressors, and have subsequently become a commercially significant abalone species in China [22]. The present study employs the Lvpan abalone as an experimental model and aimed to define the essential amino acid requirements of this hybrid abalone by analyzing the amino acid composition of their somatic tissues and then applying the ideal protein paradigm. Furthermore, this study explores the effects of supplemental lysine in low-protein diet formulations on a range of physiological and performance metrics in Lvpan abalone, including growth indices, apparent feed digestibility, amino acid metabolism, and nitrogen excretion rates (NERs). The primary objective is to determine the extent to which lysine, acting as the principal limiting amino acid, can facilitate a reduction in dietary protein levels for Lvpan abalone. The ultimate aim was to provide both a theoretical framework and practical recommendations for the future application of low-protein compound feeds in the culture of this hybrid species.

## Methods

### Animal ethics approval

All the animal care and handling procedures in this study were approved by the Animal Care Committee of the Xiamen University.

### Formulation design and feed preparation

A diet containing 27% crude protein (CP) served as the control (CP27). Experimental low-protein diets were formulated by reducing the protein content by 4% (CP23) and 8% (CP19). Based on these low-protein formulations, crystalline lysine was supplemented to match the lysine concentration of the control diet, resulting in the CP23 + AA and CP19 + AA diets (Table 1). Prior to diet formulation, all raw ingredients underwent ultra–micropulverization and were subsequently passed through an 80-mesh sieve. The ingredients were accurately weighed (±0.01 g) according to their proportional representation in each formulation and combined using a stepwise mixing procedure. Y_2_O_3_ (1 g/kg diet) was included as an inert digestibility marker. Diets were formulated based on total amino acids. Approximately 20% (w/w) of distilled water was then added to the raw material mixture, followed by thorough homogenization using a mixing apparatus (HY-5; Huanyu, Inc). The resulting homogeneous and pliable mixture was transferred to a roller mill, flattened, and sectioned into flake-like segments (4 cm × 3 cm × 1 cm). These flakes were steamed for 10 min at 105°C and then desiccated at 60°C for 6 h, until the feed moisture content was reduced to below 8%. The abalone flake-based compound feed was then obtained through sieving and stored at −20°C.

**TABLE 1:** Formulation and proximate composition of the experimental diet.

Ingredients (g/kg)	27CP	23CP	19CP	23CP+AA	19CP+AA
Fish meal	55.0	35.0	15.0	35.0	15.0
Wheat gluten	65.0	45.0	25.0	45.0	25.0
Soybean meal	200.0	180.0	160.0	180.0	160.0
Kelp meal	300.0	300.0	300.0	300.0	300.0
Fish oil	10.0	10.0	10.0	10.0	10.0
Soybean oil	10.0	10.0	10.0	10.0	10.0
High-gluten flour	200.0	200.0	200.0	200.0	200.0
Corn starch	83.0	143.0	203.0	140.5	198.1
Choline chloride	5.0	5.0	5.0	5.0	5.0
Ca(H_2_PO_4_)_2_	10.0	10.0	10.0	10.0	10.0
Ethoxyquin	1.0	1.0	1.0	1.0	1.0
Y_2_O_3_	1.0	1.0	1.0	1.0	1.0
Vitamin mix^1^	20.0	20.0	20.0	20.0	20.0
Mineral mix^2^	40.0	40.0	40.0	40.0	40.0
Lysine	-	-	-	2.5	4.9
Total (g)	1000	1000	1000	1000	1000
Essential amino acids (%, dry matter)					
Lysine	1.16	0.91	0.67	1.16	1.16
Methionine	0.48	0.40	0.31	0.40	0.31
Tryptophan	0.25	0.20	0.15	0.20	0.15
Threonine	0.75	0.62	0.49	0.62	0.49
Leucine	1.68	1.36	1.06	1.36	1.06
Isoleucine	1.03	0.85	0.66	0.85	0.66
Arginine	1.35	1.12	0.90	1.35	1.35
Phenylalanine	1.10	0.91	0.71	0.91	0.71
Valine	1.13	0.92	0.72	0.92	0.72
Histidine	0.54	0.44	0.35	0.44	0.35
Proximate composition (g/kg diet)					
Crude protein	267.5	227.2	186.9	229.2	191.0
Crude lipid	39.5	37.3	35.0	37.3	34.8
Crude ash	225.7	219.4	213.4	219.4	213.1
Gross energy (kJ/g)	15.83	15.83	15.83	15.79	15.75

CP27 means a diet containing 27% crude protein. CP23 means a diet containing 23% crude protein. CP19 means a diet containing 19% crude protein. Based on the formulations of CP23 and CP19 groups, crystalline lysine was supplemented to match the lysine concentration of the diet, named as the CP23+AA and CP19+AA diets.

1 Vitamin mix (IU or mg/kg diet): vitamin A 100,000 IU; vitamin D3 320,000 IU; vitamin E 4600 IU; vitamin K, 1000 mg; biotin, 8 mg; folic acid 400 mg; vitamin B1 1500 mg; niacin, 800 mg; inositol, 12,800 mg; calcium pantothenate, 2000 mg; vitamin B2, 2800 mg; vitamin B6, 1000 mg, vitamin B12, 0.18 mg.

2 Mineral mix (mg/kg diet): NaCl, 400; MgSO_4_·7H_2_O, 6000; NaH_2_PO_4_·2H_2_O, 10,000; KH_2_PO_4_, 12,800; FeSO_4_, 1000; ZnSO_4_·7H_2_O, 360,000; CoCl_2_·6H_2_O, 2; KI, 5.4; MnSO_4_·H_2_O, 120; CuSO_4_·5H_2_O, 96; Na_2_SeO_3_·5H_2_O.

### Growth trials and feeding management

Prior to commencing the feeding trial, a sample of 24 Lvpan abalones, with an average initial weight of 20.00 ± 0.42 g, was obtained from the Jinjiang Fuda Abalone Farm. These specimens were used to determine baseline nutrient and amino acid compositions in their soft tissues. The rearing experiment was conducted within a recirculating aquaculture system (RAS-3000L; Fuda Aquaculture Systems) located at the Jinjiang Fuda Abalone Farm. Seawater was sourced from the Jinjiang coastal area (approximate coordinates: 24.68° N, 118.54° E), where it had been sand-filtered prior to use. During the trial, the water temperature was maintained at 23°C, with fluctuations kept below 1°C throughout the 84-d constant-temperature rearing period. Before the experiment, a cohort of 450 Lvpan abalones, exhibiting initial shell lengths of 55.81 ± 0.64 mm and initial body weights of 19.49 ± 0.52 g, was selected and subjected to a 2-wk acclimation period. Following acclimation, 450 Lvpan abalones were randomly assigned to 15 rectangular black cages (43 cm long × 34 cm wide × 15 cm deep, with a density of 30 abalones per cage) and then placed into corresponding seawater temperature-controlling systems. A randomized complete design was used to assign the dietary treatments to the experimental units. Each dietary treatment was randomly assigned to 3 replicate cages. Within the recirculating water temperature control system, Lvpan abalones were fed daily at 16:00, with a ration corresponding to 1%–2% of their total biomass. Uneaten feed was removed from the culture cages at 08:00 the following morning, and feces were thoroughly rinsed to maintain the water quality. Throughout the 84-d rearing experiment, the following parameters were maintained: water temperature fluctuation below 1°C, dissolved oxygen concentration exceeding 6 mg/L, salinity ranging from 30‰ to 32‰, and pH between 7.41 and 7.93. Natural lighting was used, and the mortality rate of abalone within each culture cage was continuously monitored and recorded.

### Digestion trial

Upon completion of the rearing experiment, 12 Lvpan abalones, selected for their uniform size from each treatment group, were transferred to individual rectangular aquaria (∼80 cm × 60 cm × 30 cm). Water quality parameters were carefully monitored and maintained throughout the experiment: pH 7.65 ± 0.21, water flow rate 2.0 L/min, and temperature 23 ± 1.0°C. Dissolved oxygen was maintained above 6 mg/L. A 3-d acclimation period was implemented to ensure normal feeding behavior before initiating a 7-d digestibility trial. The feeding protocol during the digestibility trial was consistent with that used in the preceding growth experiment. Feces produced by the abalone in each treatment group were collected individually 16 h after feeding. These collected feces were then filtered through a 0.04-mm pore-size silk screen, and the material retained on the filter was stored at −20°C. The methodologies employed for the determination of nutritional components in feed, feces, and abalone soft tissue were consistent with the methods detailed later in *Nutrient composition analysis* section. Yttrium (Y) quantification was performed using an inductively coupled plasma optical emission spectrometer (Agilent 5110VDV).

### Sample collection

Following the 84-d rearing experiment, all abalones were subjected to a 48-h starvation period. Subsequently, the abalones in each culture cage were counted, their shell lengths were measured, and individual weights were recorded to determine the survival rate, shell length increase, and weight gain. Twelve abalones with similar shell lengths and overall weights were then randomly selected from each experimental group for dissection. Muscle and liver tissues were excised, transferred to 2-mL cryovials, and immediately immersed in liquid nitrogen. All samples were then cryogenically stored at −80°C for subsequent analysis of enzymatic activity and nutrient composition.

### Nutrient composition analysis

The nutritional composition of both the experimental diets and abalone soft tissues was determined using established analytical procedures. Moisture content was quantified by vacuum freeze drying. After pulverizing the lyophilized samples, the total nitrogen content was initially measured. CP content was then calculated using the Kjeldahl method with a conversion factor of *N* × 6.25. Concurrently, crude lipid content was determined using Soxhlet extraction with a liquid-phase extraction system. Ash content was determined following calcination in a muffle furnace at 550°C for 8 h, according to the Association of Official Analytical Chemists (1995) methodologies [23]. Gross energy was determined using the Parr 6100 Automatic Bomb Calorimeter.

### Amino acid composition analysis

Approximately 0.3 g of the lyophilized sample was accurately weighed into a hydrolysis tube, and the weight was recorded. Eight milliliters of 6 M HCl were then added to the tube. After securely capping the tube, it was incubated in an oven at 120°C for 22 h to facilitate hydrolysis. Following hydrolysis, 4.8 mL of 10 M NaOH was added to the tube, and the volume was adjusted to 25 mL with distilled water. The resulting solution was then filtered through double-layered filter paper. After filtration, 1 mL of the clear filtrate was transferred to a 1.5-mL microcentrifuge tube and centrifuged at 15,000 × *g* for 30 min. After centrifugation, 400 μL of the supernatant was carefully decanted and transferred to a liquid chromatography vial for analysis. All amino acids were analyzed using similar methods, with a slight modification for tryptophan determination, which required alkaline hydrolysis. Initially, 8 mL of 5 M NaOH was added, followed by neutralization with 6.7 mL of 6 M HCl. Subsequent steps were as described above. Amino acid quantification was performed using an L-8900 amino acid analyzer (Hitachi).

### Determination of the leaching loss rate of feed

The initial weight of each of the 5 experimental diets, following accurate weighing, was recorded as L_0_. Triplicate samples of each diet were then sealed in 100-μm mesh nylon bags. Subsequently, these bags, each containing a diet, were deployed in triplicate within the abalone culture tanks, which were part of a recirculating water temperature control system. Environmental conditions in the tanks holding the feed samples were maintained to replicate those established during the rearing experiment, but without abalone. At 12 and 24 h, the compound feed samples were retrieved from the nylon bags. The samples were then desiccated until a constant weight was achieved, and this final weight (FW) was recorded as *L*_*t*_.

### Determination of amino acid transporter enzyme activity

For measurement, the abalone’s intestines were collected and weighed. A homogenization buffer was added at 9 times the mass of the intestines, and the tissue was disrupted using a tissue homogenizer at 4°C. After centrifugation (3000 × *g*, 10 min, 4°C), the supernatant was collected for analysis. The total protein content of the homogenate was determined using a total protein assay kit (Nanjing Jiancheng Bioengineering Institute, A045-2-2). The enzymatic activities of amino acid transaminases were individually evaluated employing commercial assay kits designed for aspartate aminotransferase (AST; BOXBIO, AKAM019M) and alanine aminotransferase (ALT; BOXBIO, AKAM006M).

### Determination of meat quality indexes

#### Water-holding capacity determination

Water-holding capacity (WHC) was estimated by measuring centrifugal loss. Centrifugal loss was determined using a slightly modified version of the method described by Tan et al. [24]. For all WHC assays, surface moisture was first removed using absorbent paper prior to sample processing. Samples were then shaped using a template to achieve dimensions of ∼1 cm × 1 cm × 1 cm, and the initial weight of the shaped sample, ∼1 g (*W*_1_), was recorded. Subsequently, samples were wrapped in double-layered filter paper and centrifuged using an Optima L-100 XP ultracentrifuge (Beckman Coulter) at 10,000 × *g* for 15 min at 4°C. Following centrifugation, samples were reweighed, and the FW was recorded as *W*_2_. Centrifugal loss was calculated using the following formula:

$$The\;centrifugal\;loss\left(\%\right)=100\times \frac{\left(W_{1}-W_{2}\right)}{W_{1}}$$

Cooking loss was measured with minor adaptations to the method described by Zhang et al. [25]. Briefly, samples were blotted dry as above, then trimmed with a manual blade to dimensions of 4 cm × 3 cm × 1 cm, and the initial weight, *W*_3_, was recorded. The prepared samples were then sealed in polyethylene bags and immersed in a 100°C water bath for 15 min. After heating, the samples were removed, the excess surface moisture was blotted dry using absorbent paper, and the FW was recorded as *W*_4_. Cooking loss was then calculated using the following equation:

$$The\;cooking\;loss\left(\%\right)=100\times \frac{\left(W_{3}-W_{4}\right)}{W_{3}}$$

Thawing loss was measured following a modified procedure based on the study by Honikel [26]. Briefly, frozen samples were removed from a −20°C freezer, blotted dry as above, and then the initial weight was recorded as *W*_5_. Samples were then sealed in polyethylene bags and thawed in a 4°C refrigerator for 6 h. After thawing, samples were removed, the excess surface moisture was carefully blotted dry, and the FW was promptly recorded as *W*_6_. Thawing loss was then calculated using the following formula:

$$The\;thawing\;loss\left(\%\right)=100\times \frac{\left(W_{5}-W_{6}\right)}{W_{5}}$$

#### Texture profile analysis

Texture profile analysis (TPA) was performed on cylindrical specimens (1.27 cm diameter, 1.00 cm height) from the central region of prepared abalone foot muscle using a texture analyzer (TA.XT.plus; Stable Micro Systems Ltd) with a flat-faced cylindrical probe (50-mm diameter). The analysis was based on the method of Zhu et al. [25], with minor modifications. The following were the specific parameters: P/50 test probe; pre-test, test and post-test speeds, 1 mm/s; compression ratio, 60%; 2 compression cycles with a 5-s interval; data acquisition rate, 400 points/s; trigger force, 5 g; and 6 replicates per sample.

#### Electronic nose analysis

Electronic nose (E-nose) analysis, with minor modifications to the method of Zhang et al. [27], was performed using a portable E-nose odor monitoring and identification system (PEN3; Airsense Analytics GmbH). The system includes 9 metal oxide semiconductor sensors, with sensor details listed in Table 2. Briefly, ∼2.00 g of finely minced abalone sample was accurately weighed and transferred to a 20-mL headspace vial. The vial was then hermetically sealed, and the headspace allowed to equilibrate at 40°C for 30 min. A programmed temperature gradient was used for analysis (sample injection rate, 200 μL/s; injection duration, 60 s; and initial column temperature, 50°C). To prevent carryover of residual volatile compounds, the sensors were immersed in deionized water for 2 min before each sample injection. All samples were analyzed in triplicate, and the data were averaged for statistical analysis.

**Table 2:** Information of 9 sensors for electronic nose.

Sensor name	Response characteristics	Reference substances
S1	Aromatic	Benzaldehyde, cinnamaldehyde
S2	Nitrogen, amines	Methylamine, dimethylamine
S3	Sulfur	Thiol, sulfide
S4	Organic acid esters, terpenes	Linalool, citral
S5	Terpenes, esters	Cis-3-hexenyl benzoate
S6	Sterols, triterpenes	1,2,3,5,6-Pentathiepane
S7	Hydrocarbons, oxygen derivatives	Cyclohexanol
S8	Amines	Trimethylamine
S9	Furan	Furanone

### Calculation formulas and statistical analyses

The calculation formulas for protein efficiency ratio (PER), protein retention value (PRV), NER, and apparent digestibility coefficient (ADC) are presented below:

$$PER\left(\%\right)=100\times \frac{\left(W_{t}-W_{0}\right)}{W_{f\;}\times \frac{L_{d4}+L_{d16}}{2}\times {\;P}_{f}\times \frac{L_{p4}+L_{p16}}{2}}$$

$$PRV\left(\%\right)=100\times \frac{\left(W_{t}\times {\;P}_{t}-W_{0}\times {\;P}_{0}\right)}{W_{f\;}\times \frac{L_{d4}+L_{d16}}{2}\times {\;P}_{f}\times \frac{L_{p4}+L_{p16}}{2}}$$

$$NER\left(mg/kg\cdot h\right)=\frac{\left(N_{16}-N_{0}\right)}{16\times W}\times V$$

$${ADC}_{N}\left(\%\right)=\left(1-\frac{I_{R}}{I_{F}}\times \frac{N_{F}}{N_{R}}\right)\times 100$$

In these formulas: *W*_0_, *W*_t_, and *W*_f_ represent the initial body weight (g), final body weight (g), and weight of feed administered (g) to the abalone, respectively; *P*_0_, *P*_t_, and *P*_f_ denote the initial protein content (g) in the abalone, the final protein content (g) in the abalone, and the protein content (g) in the feed, respectively; L_d4_ and L_d16_ correspond to the leaching rates of the diet after 4 and 16 h, respectively; L_p4_ and L_p16_ represent the leaching rates of protein from the diet after 4 and 16 h, respectively; N_16_ indicates the nitrogen concentration in the water column 16 h postfeeding; N_0_ signifies the baseline nitrogen concentration in the water column of the control group 16 h postfeeding; V represents the water volume (L); W represents the total biomass of abalone (kg). *I*_*R*_ represents the indicator content in the feed, and *I*_*F*_ represents the indicator content in the feces. *N*_*R*_ represents the nutrient content in the feed, and *N*_*F*_ represents the content of that nutrient in the corresponding feces.

Normality (Shapiro–Wilk) and homogeneity (Levene's) were verified before data analysis. Outliers were identified using the Grubbs’ test (*α* = 0.05) and removed. Multiple comparisons were performed using Duncan’s test following a 1-way analysis of variance. Statistical analyses were performed using SPSS, version 26.0 (IBM). Experimental data are expressed as mean ± SD, and *P* < 0.05 was used as the significant difference level.

## Results

### Amino acid content in body composition and the ratio of each essential amino acid based on the ideal protein concept in Lvpan abalone

**FIGURE 1:**
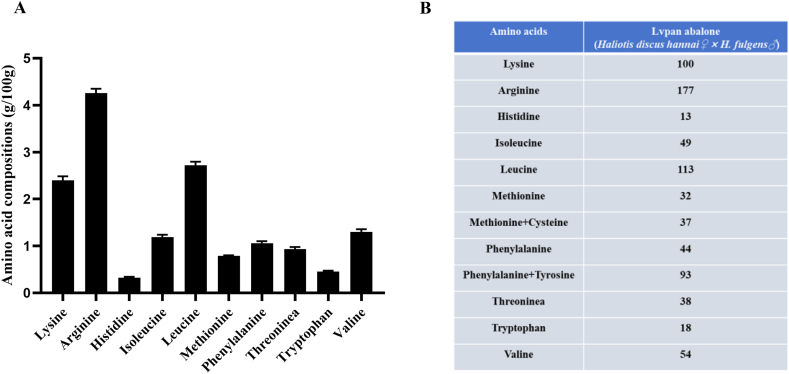
The essential amino acid composition and calculated ratios of each essential amino acid to lysine of Lvpan abalone. (A) The essential amino acid compositions of the whole soft body of Lvpan abalone. (B) Calculation of the ratio of 10 essential amino acids to lysine in Lvpan abalone using the “ideal protein” concept (%). Note: Values are means ± SD (*n* = 6).

The essential amino acid composition of Lvpan abalone is shown in Figure 1A. The most abundant essential amino acids were arginine (4.25 ± 0.09), leucine (2.71 ± 0.07), and lysine (2.40 ± 0.08). Based on the “ideal protein” concept, using lysine as the primary limiting amino acid, the estimated ideal ratios of the 10 essential amino acids for Lvpan abalone are presented in Figure 1B. Using lysine as the reference, the dietary ratios of methionine, tryptophan, and threonine to lysine were 0.32, 0.18, and 0.38, respectively.

### The leaching loss of dry matter and protein in compound feeds

**FIGURE 2:**
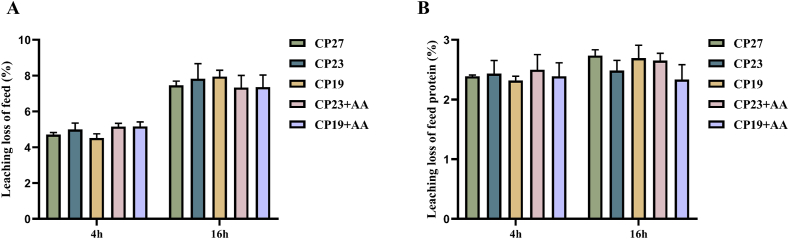
Effect of different immersion time on the leaching loss rate of feed and feed protein. (A) The leaching loss of feed. (B) The leaching loss of feed protein. Note: CP27 means a diet containing 27% crude protein. CP23 means a diet containing 23% crude protein. CP19 means a diet containing 19% crude protein. Based on the formulations of CP23 and CP19 groups, crystalline lysine was supplemented to match the lysine concentration of the diet, named as the CP23+AA and CP19+AA diets.

The effects of immersion time on the leaching rate and protein leaching rate from experimental feeds are shown in Figure 2. The leaching rates of all 5 experimental feeds increased with immersion time, averaging 4.90% ± 0.44% after 4 h and 7.58% ± 0.91% after 16 h. There were no significant differences (*P* > 0.05) in leaching rate between the 5 feeds at either time. Protein leaching rates averaged 2.40% ± 0.27% after 4 h and 2.58% ± 0.31% after 16 h. Protein leaching rates were not significantly affected by immersion time and did not differ significantly between feeds (*P* > 0.05).

### Effect of lysine supplementation in low-protein feeds on the growth and survival of Lvpan abalone

**FIGURE 3:**
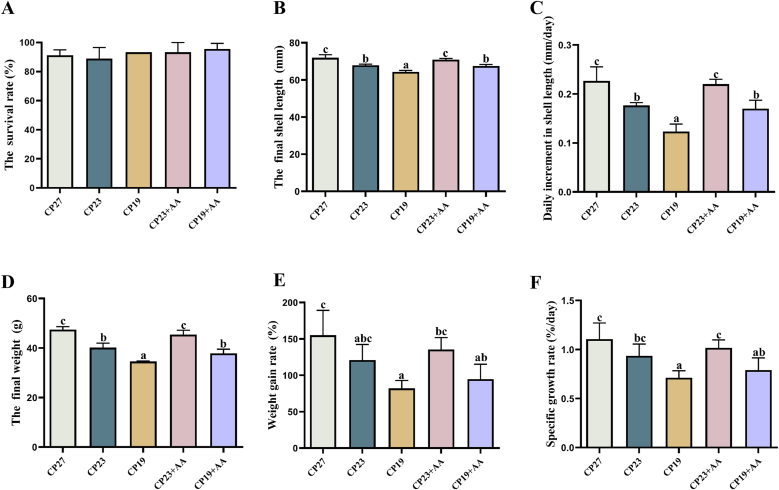
Effect of adding lysine in low-protein diet on the growth performance of Lvpan abalones. (A) The survival rate; (B) the final shell length; (C) daily increment in shell length; (D) the final weight; (E) weight gain rate; and (F) specific growth rate. Note: CP27 means a diet containing 27% crude protein. CP23 means a diet containing 23% crude protein. CP19 means a diet containing 19% crude protein. Based on the formulations of CP23 and CP19 groups, crystalline lysine was supplemented to match the lysine concentration of the diet, named as the CP23+AA and CP19+AA diets. Different superscripts on the error line indicate significant differences (*P* < 0.05).

The effect of lysine supplementation in low-protein diets on Lvpan abalone growth and survival is shown in Figure 3. Survival was above 80% in all 5 treatments, with no significant differences (*P* > 0.05). No significant differences were observed in final shell length (FSL), daily shell length increase (DISL), FW, weight gain rate (WGR), and specific growth rate (SGR) between the CP23 + AA and CP27 groups, as well as the CP23 and CP19 + AA groups (*P* > 0.05). Lvpan abalone-fed lysine-supplemented diets (CP23 + AA and CP19 + AA) had greater FSL, DISL, FW, WGR, and SGR than those fed nonsupplemented diets (CP23 and CP19), with significant differences for FSL, DISL, and FW (*P* < 0.05).

### Effect of lysine supplementation in low-protein feeds on apparent digestibility, PER, protein retention ratio, and nitrogen excretion in Lvpan abalone

**FIGURE 4:**
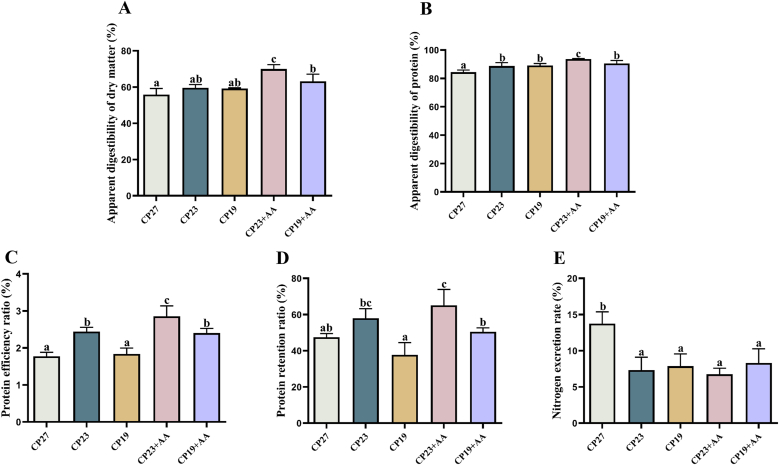
Effect of adding lysine in low-protein diet on the apparent digestibility, protein metabolism and nitrogen excretion of Lvpan abalones. (A) Apparent digestibility of dry matter; (B) Apparent digestibility of protein; (C) Protein efficiency ratio; (D) Protein retention rate; (E) Nitrogen excretion rate. Note: CP27 means a diet containing 27% crude protein. CP23 means a diet containing 23% crude protein. CP19 means a diet containing 19% crude protein. Based on the formulations of CP23 and CP19 groups, crystalline lysine was supplemented to match the lysine concentration of the diet, named as the CP23+AA and CP19+AA diets. Different superscripts on the error line indicate significant differences (*P* < 0.05).

The effect of lysine supplementation in low-protein diets on apparent digestibility, PER, protein retention ratio, and nitrogen excretion in Lvpan abalone is shown in Figure 4. Both apparent dry matter and protein digestibility in the CP23 and CP19 groups increased as dietary protein decreased. The apparent digestibility of protein in the CP23 and CP19 groups was significantly higher than that in the CP27 group (*P* < 0.05). Lysine supplementation (CP23 + AA) significantly increased apparent dry matter and protein digestibility compared with all other diets (*P* < 0.05). The CP23 group had significantly greater protein efficiency and protein retention than the CP27 and CP19 groups (*P* < 0.05), but there was no significant difference between the CP27 and CP19 groups (*P* > 0.05). Lysine supplementation (CP23 + AA and CP19 + AA) significantly increased protein efficiency compared with the unsupplemented groups (CP23 and CP19) (*P* < 0.05), and CP19 + AA significantly increased protein retention compared with CP19 (*P* < 0.05), with a nonsignificant trend for CP23 + AA compared with CP19 (*P* > 0.05). Nitrogen excretion was significantly greater in the CP27 group than in the other 4 groups (*P* < 0.05), with no significant differences among the remaining groups (*P* > 0.05).

### Effect of lysine supplementation in low-protein feeds on hepatic aminotransferase activity in Lvpan abalone

**FIGURE 5:**
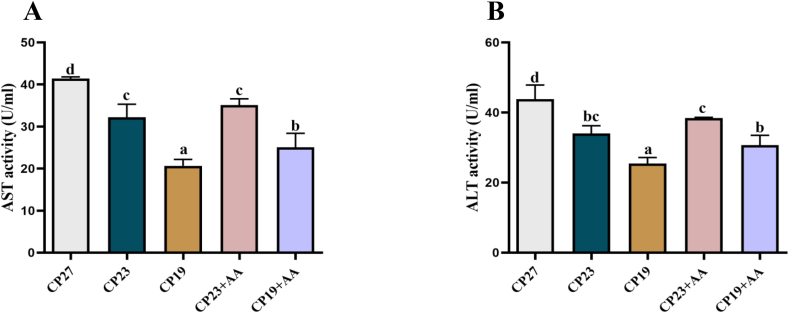
Effect of adding lysine in low-protein diet on the transaminase of liver in Lvpan abalones. (A) Aspartate aminotransferase (AST); (B) Alanine aminotransferase (ALT). Note: CP27 means a diet containing 27% crude protein. CP23 means a diet containing 23% crude protein. CP19 means a diet containing 19% crude protein. Based on the formulations of CP23 and CP19 groups, crystalline lysine was supplemented to match the lysine concentration of the diet, named as the CP23+AA and CP19+AA diets. Different superscripts on the error line indicate significant differences (*P* < 0.05).

The effect of lysine supplementation in low-protein diets on hepatic samples' aminotransferase activity from Lvpan abalone is shown in Figure 5. Hepatic AST and ALT activities in Lvpan abalone decreased significantly (*P* < 0.05) with reduced dietary protein. Lysine supplementation in the CP23 + AA group increased transaminase activity compared with the CP23 group, although this was not statistically significant (*P* > 0.05). Transaminase activity was significantly greater in the CP19 + AA group than in the unsupplemented CP19 group (*P* < 0.05).

### Effect of lysine supplementation in low-protein feeds on muscle nutrient composition in Lvpan abalone

**FIGURE 6:**
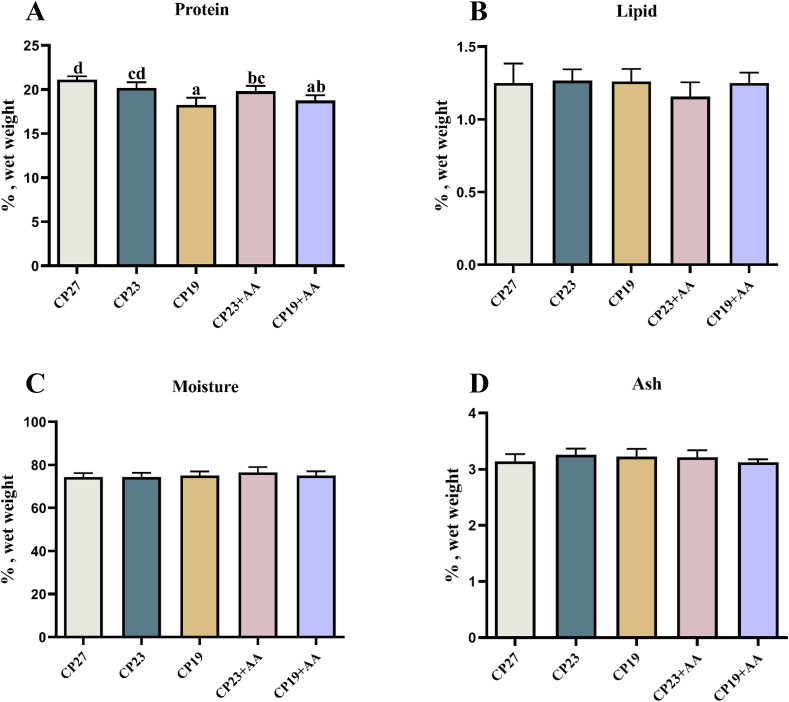
Effect of adding lysine in low-protein diet on the nutrient composition of muscle in Lvpan abalones. (A) Proteins; (B) lipid; (C) moisture; and (D) ash. Note: CP27 means a diet containing 27% crude protein. CP23 means a diet containing 23% crude protein. CP19 means a diet containing 19% crude protein. Based on the formulations of CP23 and CP19 groups, crystalline lysine was supplemented to match the lysine concentration of the diet, named as the CP23+AA and CP19+AA diets. Different superscripts on the error line indicate significant differences (*P* < 0.05).

The effect of lysine supplementation in low-protein diets on muscle nutrient composition in Lvpan abalone is shown in Figure 6. Muscle protein content decreased proportionally with reduced dietary protein and was significantly less in the CP19 group than in the CP27 group (*P* < 0.05). Lysine supplementation (CP23 + AA and CP19 + AA) increased muscle protein content compared with the unsupplemented groups (CP23 and CP19), although differences were not significant (*P* > 0.05). There were no significant differences in other nutrient components (*P* > 0.05).

### Effect of lysine supplementation in low-protein feeds on WHC of meat in Lvpan abalone

**FIGURE 7:**
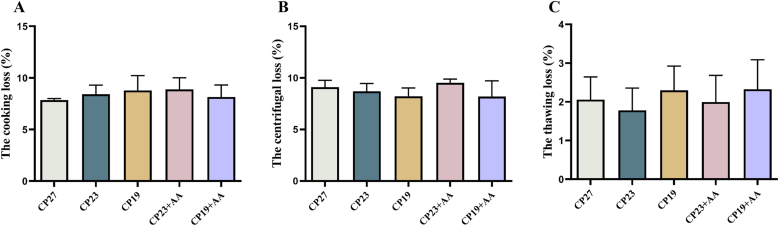
Effect of adding lysine in low-protein diet on the water-holding capacity of muscle in Lvpan abalones. (A) The cooking loss; (B) The centrifugal loss; (C) The thawing loss. Note: CP27 means a diet containing 27% crude protein. CP23 means a diet containing 23% crude protein. CP19 means a diet containing 19% crude protein. Based on the formulations of CP23 and CP19 groups, crystalline lysine was supplemented to match the lysine concentration of the diet, named as the CP23+AA and CP19+AA diets.

The effect of lysine supplementation in low-protein diets on muscle WHC in Lvpan abalone is shown in Figure 7. There were no significant differences in cooking loss, centrifugal loss, or thawing loss among the treatments (*P* > 0.05).

### Effect of lysine supplementation in low-protein feeds on meat textural parameters of Lvpan abalone

**FIGURE 8:**
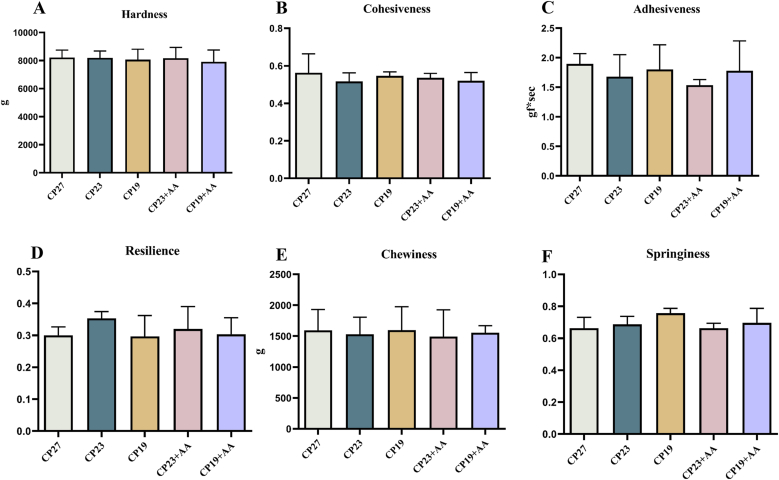
Effect of adding lysine in low-protein diet on texture parameters of muscle in Lvpan abalones. (A) Hardness; (B) cohesiveness; (C) adhesiveness; (D) resilience; (E) chewiness; and (F) springiness. Note: CP27 means a diet containing 27% crude protein. CP23 means a diet containing 23% crude protein. CP19 means a diet containing 19% crude protein. Based on the formulations of CP23 and CP19 groups, crystalline lysine was supplemented to match the lysine concentration of the diet, named as the CP23+AA and CP19+AA diets.

The effect of lysine supplementation in low-protein diets on meat texture parameters in Lvpan abalone is shown in Figure 8. There were no significant differences among the treatments in texture parameters (hardness, cohesiveness, adhesiveness, resilience, chewiness, and elasticity) (*P* > 0.05).

### Effect of lysine supplementation in low-protein feeds on meat flavor in Lvpan abalone

**FIGURE 9:**
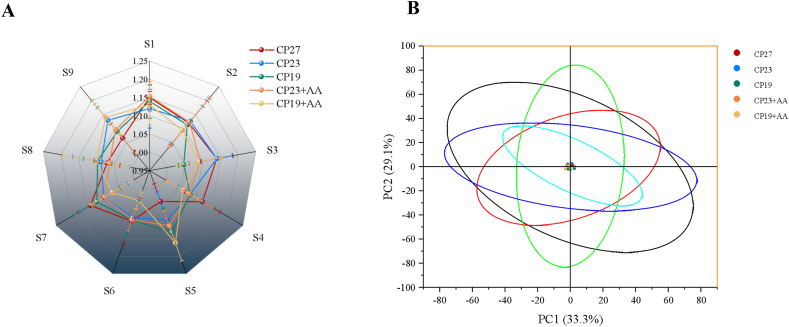
Effect of adding lysine in low-protein diet on the E-nose analysis of muscle in Lvpan abalones. Flavor radar chart; and (B) principal component analysis. Note: CP27 means a diet containing 27% crude protein. CP23 means a diet containing 23% crude protein. CP19 means a diet containing 19% crude protein. Based on the formulations of CP23 and CP19 groups, crystalline lysine was supplemented to match the lysine concentration of the diet, named as the CP23+AA and CP19+AA diets.

Figure 9 shows the E-nose response patterns to muscle samples from Lvpan abalones fed lysine-supplemented low-protein diets. There were no significant differences in responses from the 9 E-nose sensors (S1–S9) among the 5 treatments (*P* > 0.05). Principal components PC1 and PC2 accounted for 33.3% and 29.1% of the total variance, respectively, with a cumulative contribution of 62.4%.

## Discussion

### An essential amino acid balance model based on the ideal protein concept in Lvpan abalone

Abalone growth depends on amino acids rather than intact proteins, making precise regulation of essential amino acid profiles in feeds essential to maximize protein deposition and growth efficiency [28]. Our analysis revealed that lysine, arginine, and leucine were the predominant essential amino acids in Lvpan abalone tissue, consistent with findings in red abalone [29] and blacklip abalone [30]. Based on these amino acid profiles, we derived lysine-based ideal ratios for 10 essential amino acids (methionine: 0.32; tryptophan: 0.18; and threonine: 0.38) for Lvpan abalone. These ratios provide a valuable guideline for feed formulation, pending further species-specific amino acid requirement studies.

### Effect of lysine supplementation in low-protein feeds on the growth performance of Lvpan abalone

Low-protein diets are designed to reduce CP content while maintaining animal performance and product quality through precise supplementation with synthetic amino acids. This approach decreases protein ingredient use, feed costs, and nitrogen emissions [31]. Reduced protein diets generally need crystalline amino acid supplementation to balance essential amino acid profiles and prevent growth limitations caused by deficiencies in limiting amino acids [32]. Lysine is a crucial basic amino acid for aquatic animal growth and immunity [5] and is the primary limiting amino acid in abalone protein sources [28], with particularly large deficiencies in plant-based feeds. Our results showed that reduced dietary protein impaired growth performance in Lvpan abalone, and that lysine supplementation in low-protein diets only partially reversed this effect. Salem et al. [6] achieved 6% protein reduction (32%–26%) in channel catfish (*Ictalurus punctatus*) feeds through lysine balancing without compromising growth performance. Zhou et al. [33] reduced protein by 3.2% (33.5%–30.3%) in Nile tilapia (*O. niloticus*) feeds with methionine, lysine, tryptophan, and threonine supplementation based on ideal protein concepts. Gaylord and Barrows [34] found that rainbow trout (*O. mykiss*) maintained growth performance with a 4.5% protein reduction (46%–41.5%) when supplemented with lysine, methionine, and threonine. Cheng et al. [13] showed that 3%–5% protein reductions in rainbow trout (*O. mykiss*) feeds did not affect growth when essential amino acids were adequately supplemented. Collectively, these studies show that moderate protein reduction with limiting amino acid supplementation can maintain growth in aquatic species, but none examined protein levels low enough to induce essential amino acid limitations. Our data showed significant growth depression in Lvpan abalone with 19% dietary protein, even with lysine balance. Reduced growth in the CP19 and CP19 + AA groups likely resulted from insufficient nonessential amino acid synthesis when dietary protein fell to 19%, ultimately limiting protein synthesis. This deficiency may have limited the growth performance of abalone in these treatment groups, despite lysine supplementation. The lack of sufficient levels of other essential amino acids may have constrained protein synthesis and the efficient utilization of lysine. Salem et al. [6] reported that growth did not recover with lysine/methionine supplementation after an 8% protein reduction, suggesting that multiple amino acid limitations occur when protein levels fall below critical thresholds. Further research is needed to determine the optimal balance of all essential amino acids in low-protein diets for abalone.

### Effects of lysine supplementation in low-protein feeds on apparent digestibility, nitrogen excretion, and amino acid metabolism of Lvpan abalone

Apparent dry matter digestibility reflects the percentage of dry matter in the diet that is not excreted in the feces, providing an indication of the overall digestibility of the major dietary components, including protein, lipid, carbohydrate, and ash [35]. Apparent protein digestibility reflects the percentage of dietary protein that is not excreted in the feces, indicating the efficiency with which the animal can digest and absorb dietary protein [36]. Our results showed that reducing dietary protein increased apparent dry matter and protein digestibility in Lvpan abalone, and that lysine supplementation further improved these. Similarly, Cheng et al. [13] reported that lysine supplementation of low-protein diets significantly increased apparent CP digestibility in rainbow trout (*O. mykiss*). Collectively, these studies indicate that supplementing lysine in low-protein diets increases apparent digestibility, thus optimizing nutrient absorption in aquatic species.

Nitrogenous waste is produced during protein metabolism and excreted into the environment, contributing to pollution [37]. Reducing dietary protein can decrease environmental pollution from aquatic animal nitrogen excretion. Our results showed that dietary protein restriction significantly reduced nitrogen excretion in Lvpan abalone. However, adding lysine to a low-protein diet does not affect NERs. Viola et al. [38] found that lysine supplementation of low-protein diets reduced nitrogen excretion by ∼20% in carp (*Cyprinus carpio*). These differences may be due to the separate components of fecal and urinary nitrogen: fecal nitrogen (undigested dietary nitrogen) is protein-level dependent [39], whereas urinary nitrogen represents deaminated amino acid residues from protein metabolism and is affected by dietary amino acid balance [40]. In fin fish, nitrogen is primarily excreted as ammonia via the gills and feces. Although the primary excretory product in abalone and its regulation by diet are not fully understood, it is possible that our study was not long enough and/or not sensitive enough to detect significant differences in NERs among the treatments. Furthermore, the complex interplay between fecal and urinary nitrogen, as well as the effects of dietary amino acid balance on nitrogen excretion, warrants further investigation in abalone.

Transaminases, particularly hepatic AST and ALT, are key to amino acid metabolism in aquatic species, and their activity is used to indicate protein metabolism [41]. Reducing dietary protein decreased AST and ALT activities in abalone, suggesting that excess protein causes hepatic stress and damage. Lysine supplementation moderately increased transaminase activity compared with the unsupplemented low-protein groups, but remained below CP27 levels, indicating partial restoration of hepatic function while maintaining reduced metabolic stress.

### Effects of lysine supplementation in low-protein feeds on the meat quality of Lvpan abalone

Muscle nutritional composition is a key indicator of abalone meat quality [42]. Protein content largely determines the nutritional value of abalone body tissue because of its high-quality protein [43]. Our results showed that muscle protein decreased in abalone-fed reduced dietary protein levels. Similar findings have been reported for giant grouper (*Epinephelus lanceolatus*) [44], hybrid catfish (*Clarias batrachus* × *Clarias gariepinus*) [45], *Colossoma macropomum* (Cuvier) [46], and gilthead sea (*Sparus aurata*) [47], which showed positive correlations between dietary protein level and protein deposition. However, other studies have found that dietary protein level did not affect body protein content in channel catfish (*I. punctatus*) [48] or Florida red tilapia (*Oreochromis aureus* × *Oreochromis mossambicus*) [49]. Lysine supplementation only slightly increased muscle protein in Lvpan abalone. Niu et al. [50] and Sardar et al. [51] reported that lysine supplementation significantly increased body protein in golden pompano (*Trachinotus ovatus*) and other fish species.

WHC is the ability of the muscle to retain moisture during mechanical stress, suspension, or heating [52]. WHC is an important indicator of meat quality because it affects texture, mouthfeel, and sensory properties [53]. Cooking loss, centrifugal loss, and thawing loss typically have an inverse relationship with WHC [54]. In this study, neither low-protein diets nor lysine supplementation had any significant effect on cooking, centrifugal, or thawing losses in abalone muscle, indicating that WHC characteristics were not altered. However, other studies have suggested that moderate protein elevation can increase WHC in abalone, possibly because it reduces oxidative damage [55]. There is limited research on the relationship between aquatic meat ultrastructure and WHC, but myofibrillar volume changes due to alterations in fiber spacing may affect cooking losses [56]. Abalone muscle is known for its exceptional toughness, a textural attribute that is as important as a flavor [57]. TPA measures several parameters, including hardness, cohesiveness, adhesiveness, resilience, chewiness, and elasticity [58]. In this study, neither reduced dietary protein nor lysine supplementation significantly affected these parameters in abalone muscle. Aquatic muscle texture is generally determined by genetic factors, diet composition, and rearing methods [59]. Our results suggest that dietary protein manipulation did not alter abalone texture. In contrast, broad bean meal supplementation significantly improved muscle firmness in grass carp (*Ctenopharyngodon idella*), tripling its market value [60]. Although diet-related texture changes have not been reported in abalone, water temperature can significantly affect muscle texture. Porturas et al. [61] found that seasonal softening of the Pacific abalone (*H. discus hannai*) texture correlated with collagen content. Other studies suggest that abalone texture is determined by myofibril:collagen fibril ratios and ultrastructure [62].

In addition to nutritional and textural parameters, flavor significantly affects consumer preference. Flavor development in aquatic animals depends on several factors, including diet and environmental conditions [63]. E-nose technology uses artificial sensory arrays to mimic mammalian olfaction. E-noses are widely used as analytical tools in the food industry to assess meat quality and profile volatile compounds [64]. E-nose analysis showed no significant differences in sensor response (S1–S9) between the dietary treatments, indicating similar flavor profiles. Principal component analysis showed a cumulative variance for PC1 and PC2 of 62.4% (<80%), which was not sufficient to distinguish between the samples. Smit et al. [65] found that flavor varied with diet in South African abalone (*Haliotis midae*)-fed *Ulva*, kelp, or formulated feeds. Chiou and Lai [66] also reported large flavor differences in small abalone (*H. diversicolor*)-fed formulated diets compared with *Gracilaria*.

In conclusion, this study calculated the ratio of essential amino acids to lysine (the first-limiting amino acid) based on the ideal protein concept in Lvpan abalone. We suggest that these amino acid ratios could guide feed formulation for this species when individual amino acid requirements have not been determined. Furthermore, the trial showed that growth performance, apparent digestibility, and muscle quality (nutrient composition, WHC, texture, and flavor) were maintained with a 4% protein reduction in lysine-supplemented diets, while also decreasing nitrogen excretion. However, an 8% protein reduction had negative effects that lysine-supplemented diets could not prevent. This study calculated the ratio of essential amino acids to lysine and evaluated the extent to which dietary protein can be reduced by lysine optimization in Lvpan abalone, providing a basis for developing low-protein aquafeeds for abalone.

## Author contributions

The authors’ responsibilities were as follows – Y-BM: conceptualization, investigation, data curation, writing—original draft; W-GZ: methodology, formal analysis, visualization; C-XA: validation, resources; S-TL: software, investigation; XL: supervision, project administration; W-WY (corresponding author): funding acquisition, writing—review and editing; C-HK (corresponding author): conceptualization, supervision, writing—review and editing; and all authors: have read and approved the final manuscript.

## Data availability

Detailed data access procedures are available upon request.

## Funding

This work was supported by grants from National Key R&D Program of China (2024YFD2401704), National Natural Science Foundation of China (U22A20530), Seed Industry Innovation and Industrialization in Fujian Province (No.2021FJSCZY02), Earmarked Fund for CARS (No. CARS-49). Thanks for the support from the Germplasm resources sharing platform of aquatic species in Fujian Province.

## Conflict of interest

The authors report no conflicts of interest.
